# On Rhinitis

**Published:** 1887-06

**Authors:** W. J. Penny

**Affiliations:** Assistant-Surgeon to the Bristol General Hospital


### ON RHINITIS.

BY

W. J. PENNY, F.R.C.S.,

*Assistant-Surgeon to the Bristol General Hospital.*

---

Rhinitis is a common affection which causes much discomfort, so that it deserves more attention than is generally accorded to it. Acute rhinitis, or the common catarrh, is unfortunately known to all; but the cases I propose to refer to, and which come more under the province of the surgeon, are those in which there is some more or less permanent lesion of the structures of the nose, and which in many cases require mechanical treatment. These cases may be roughly subdivided into those of:

1. Irritable vascular hypertrophy of the mucous membrane.
2. Chronic fibroid thickening of the membrane.
3. Chronic catarrhal rhinitis.
4. Ulcerative rhinitis
5. Polypoid rhinitis.
6. Arthritic and osteitic, combined with periosteitic rhinitis.
7. Tubercular rhinitis.
8. Lupoid rhinitis.

A large majority of these are caused by neglected colds; traumatisms; an unwholesome state of the atmosphere, depending either on the presence of chemically or mechanically irritating agents, or germs; various constitutional conditions, notably struma and syphilis, sometimes gout and rheumatism. They are mainly kept up by changes of atmospheric conditions, injudicious diet, especially alcoholic excesses—and in these cases a very small quantity means excess; in some cases, lack of cleanliness, it may be meddlesome surgery, deformities of the parts, and other causes too numerous to mention.

In these cases the pathological change is principally confined to the lower or respiratory part of the nose; in some few instances the whole of the interior of the nose is affected. In the respiratory area, according to Panas, there is a greater development of sub-mucous tissue, which permits inflammatory infiltration more readily than the upper or olfactory part, in which the mucous membrane is thinner and more directly connected with the periosteum.

The first group of cases, those of irritable vascular hypertrophy, are not quite so common as the chronic hypertrophic. They occur chiefly in neurotic subjects. The lesion is characterised by an irritable, inflamed, almost abraded-looking, thickening of the turbinated bones and septum. The usual symptoms of nasal stenosis are present, accompanied by sneezing, irritable discharge, and one or more of the symptoms of reflex disturbance in the reflex area described by Morell Mackenzie; it may be, asthma, irritable cough, weak voice, or even bronchitis. Hay fever and allied disorders are liable to supervene on slight provocation. These cases should be treated by sedative remedies; soothing injections, such as warm boracic lotion, poppy-head lotion, bathing the nose externally with the same, gargling the throat; and after this is done, anointing the nose inside and out with some sedative ointment—vaseline or lanolin, impregnated with cocaine, acetate of lead, belladonna, morphia, or eucalyptus. Powders I have found, as a rule, do more harm than good; they absorb a certain amount of the watery parts of the secretion, and the rest is left as dried cakes along the sides of the nose, and causes further irritation.

Chronic fibroid thickening, or chronic hypertrophic rhinitis, in many cases is characterised by the absence of other symptoms than nasal stenosis and mouth breathing; in others there may be symptoms referred to the pharynx, larynx, or bronchial tubes; but, as a rule, in this class the symptoms are anæsthetic, in contradistinction to the last class, in which hyperæsthesia is found. The patient complains of feeling stuffed up, and a special liability to catch cold. The swelling covering the turbinated bones and septum looks indolent, feels firm, is painless, and does not become congested when touched with a probe. For these cases I have found dilatation with bougies useful: this I believe promotes absorption of the inflammatory material. A solution of chromic acid, applied after the parts are well douched and dried, accelerates the cure.

I will quote one case.

H. N., æt. 33, a groom, for seven or eight years had complained of mouth breathing and a feeling of being stuffed up. He had consulted several medical men, one of whom had told him there was no polypus, and nothing much the matter; and another had ordered some snuff. I found that his nose was almost entirely obstructed; he could get a little air through with difficulty, and a small probe could hardly be passed along the floor of the nose. He was ordered injections, and, as he got better, snufflings of cold water. After the injection the nose was well dried, and then a solution of chromic acid, 10 grains to the ounce, painted over the septum and turbinated bones. After a few weeks of this treatment with a tonic, and occasional catheterisations, he was practically cured. In this case there was no history of constitutional taint, but the mischief apparently arose from a series of colds.

The next class of case, chronic catarrhal rhinitis, is characterised by an increased secretion from the nose. In some instances the discharge is thin, and almost serous in character; in others, of thick inspissated mucus. In some instances there is a little thickening of the membrane, in others apparently atrophy, and also of the bones beneath. Some of the cases, if neglected, run into the next class, that of ulcerative rhinitis; but the catarrhal rhinitis proper presents no macroscopical ulceration. For this class of case powders are useful; but before being used, any thick mucus should be cleared away by alkaline injections. Among powders, Ferrier's snuff, and various combinations of boracic acid, or acacia with tannin, are especially useful. Iodoform, if there is any smell, may be added with advantage to the preceding snuffs.

Ulcerative rhinitis—sometimes the result of a long-continued catarrhal rhinitis, or traumatisms, or the lodgment of foreign bodies, sometimes of gonorrhœa or tuberculosis—is intensified by a strumous or syphilitic habit; and in some, indeed, no other cause can be found. To this class also belong cases of ozæna. All ulcerative cases are not accompanied by ozæna, and ozæna is not necessarily accompanied by ulceration. The worst cases of ozæna depend on a certain amount of caries of the bone; but others may be due simply to the accumulation and putrefaction of the secretions of the nose.

These cases are best treated by copious douches, and ointment of some antiseptic material, of which I have found the following formula especially useful :

Iodoform, gr. x.

Ol. Eucalyptol, ʒ j.

Pulv. Acid. Boracic, ʒ j.

Ol. Sassafras Ethereal, ♏ x.

Vaseline ad. ℥ j.

A young man came under my care at the General Hospital, who stated that for years he had suffered from a discharge from his nose—this was not offensive; but he had anosmia, and also some loss of ordinary sensation. Two or three times during the day mucus accumulated, and he blew his nose to show me. From each nostril a mass of hard mucus was expelled, the size of a large filbert—a most sickening sight. The affection commenced with a cold, as he says, after an attack of measles followed by low fever. There was no history of syphilis or other marked constitutional affection. On examination the cavities of the nose were found large, the entrance of air free; but the mucous membrane in places was abraded and ulcerated, with that peculiar asthenic type of congestion seen sometimes surrounding ulcers of the leg. He had consulted several medical men for this complaint, all of whom had treated the matter lightly, but ordered him a powder and told him he would be all right.

Copious douches of warm water were prescribed, and a paint of chromic acid—10 gr. to ℥ j. water. A fortnight later he came, saying he had had a hæmorrhage from the nose. Since the last visit he had been using the paint, and his nose had been feeling more stuffy; the bleeding relieved this sensation. He was now ordered to continue the injections, and to use an ointment of boracic acid, iodoform and eucalyptus. The condition rapidly improved, and he is now on the high road to recovery.

To this class also belong cases of snuffles in children with inherited syphilis; local as well as general treatment should be carried out.

Cases which I have called polypoid rhinitis are not infrequently seen. They apparently depend upon some chronic irritation, and are characterised by a general thickening of the membrane, which has the appearance of diffused mucous polypus. Some of these cases come under the class described by Morell Mackenzie as sessile polypi, and Schech as polypoid vegetations: the latter also states that it is very difficult to draw a hard and fast line between hypertrophies of the mucous membrane and real neoplasms. What I wish to call attention to is, the fact that a rhinitis exists differing from the hypertrophic in the lack of firmness, and from the irritable vascular rhinitis by the lack of vascularity and hyperæsthesia. It has somewhat the appearance presented by the synovial membrane in pulpy swellings of joints. This condition is rarely found over the whole surface, but is frequently combined with a congestive thickening of the rest of the membrane, and is best treated by scarification and removal of any particularly thickened parts. Careful after-treatment must be carried out; and here again chromic acid is useful, and in some cases astringent ointments or powders.

There is another class of cases due to thickening of the bone and periosteum or perichondrium, which, when not dependent on injury, are due to some constitutional taint, chiefly syphilis and struma. These cases are recognised with difficulty, until some serious mischief is caused, when diagnosis is easy. Treatment should be carried out on general principles.

In old-standing cases of hypertrophic rhinitis, with a certain amount of thickening of the bone and periosteum, which does not subside after the treatment previously described, I have found a little oleate of mercury (10 p.c.), applied both to the interior and exterior of the nose, useful.

Tubercular or lupoid rhinitis is rare. I have not seen a well-marked case of the former; but at the present time a girl with lupoid rhinitis is under my care at the Hospital. In this case both alæ are destroyed, and the lining membrane is so thickened that the lumen is almost obliterated. The membrane presents the red tubercular appearance characteristic of lupus in other parts, and the treatment should be carried out on the same lines.

Professor Bosworth, in an interesting paper abstracted for another part of this journal, alludes to a condition which has not been previously described. He refers to bony thickenings along the sutural lines of the intra-nasal bones. This he considers is due, in the majority of cases, to traumatisms in early life, unrecognised and untreated, but which in time cause all the phenomena of reflex disturbance in the reflex area of the nose. Morell Mackenzie states that "Small exostoses can also often be perceived at the angle where the perpendicular plate of the ethmoid, the vomer and the cartilage of the septum meet one another." Professor Bosworth claims that by removing these bony growths he has cured a large number of cases of mouth breathing, asthma, hay fever, bronchitis, laryngitis, ear diseases, &c., &c. He is to be congratulated on these very favourable results.

I had occasion recently to remove a cartilaginous growth from the right nostril of a boy. In this case the top of the nose and septum were displaced to the left, and a good deal of hypertrophic rhinitis existed behind the growth on the right side. The removal of the growth was followed by marked improvement of all these symptoms. Cartilaginous tumours of the nose are rare.

In conclusion, among other methods of examination, I do not see that attention has been called to the fact that valuable indications of the condition of the nose can often be obtained from an examination of the palate through the mouth.

